# Finite Element Analysis of Trabecular-Surfaced Implants and Implant Angulation in Different Mandibular Arch Forms

**DOI:** 10.3390/jfb16090333

**Published:** 2025-09-08

**Authors:** Ahmet İlter Atay, Bahattin Alper Gültekin, Serdar Yalçın

**Affiliations:** 1 Oral Implantology Program, Institute of Graduate Studies in Health Sciences, Istanbul University, 34098 Istanbul, Turkey; 2 Faculty of Dentistry, Department of Oral Implantology, Istanbul University, 34098 Istanbul, Turkey; alperg@istanbul.edu.tr (B.A.G.);

**Keywords:** all-on-4 concept, implant surface design, mandibular arch morphology, trabecular titanium

## Abstract

Finite element analysis is commonly used to evaluate implant biomechanics, yet limited data exist on arch form and trabecular-surfaced implants. This study aimed to investigate the biomechanical impact of a designed trabecular surface compared with a standard implant surface in full-arch, four-implant-supported restorations, using two mandibular arch forms and four placement configurations. Finite element analyses were conducted under a 250-N oblique load applied at 30° to the posterior segment. The prosthesis was modeled as a titanium–acrylic hybrid structure. Stress distribution was evaluated in cortical and cancellous bones, implants, and prosthetic frameworks. Implants with a trabecular surface demonstrated lower stress concentrations in both bone and implant structures. The von Mises stress at the neck of the posterior implant decreased from 383.3 MPa (standard implant, hyperbolic arch, configuration 1) to 194.9 MPa (trabecular-surfaced implant, U-shaped arch, configuration 4). Similarly, the average maximum principal tensile stress in cortical bone reduced from 44.32 to 40.99 MPa with the trabecular design. Among placement strategies, Configuration 3 (all implants tilted distally) yielded the highest bone stress, whereas Configurations 2 and 4 provided more favorable load distribution. Stress concentrations were also higher in hyperbolic arches, whereas U-shaped arches exhibited a more uniform distribution. These findings emphasized the biomechanical advantage of the designed trabecular surface in reducing stress across bone and implant components, indicating that trabecular titanium may represent a more reliable and cost-effective alternative for clinical applications, potentially enhancing long-term stability. Independently, the arch form and placement strategy also significantly influenced load distribution. Despite assumptions such as isotropic, homogeneous, and linearly elastic material properties, and the use of a single oblique loading condition, this study offers valuable biomechanical insights such as the stress-reducing effect of the trabecular surface, the influence of three-dimensional arch anatomy on stress concentration sites, and the necessity of selecting implant configurations according to arch forms, which may inform future full-arch implant rehabilitations.

## 1. Introduction

Edentulism is a significant public health issue resulting from the loss of all natural teeth, widespread among elderly individuals [1,2]

Treatment options include conventional complete dentures, implant-supported removable prostheses, and fixed restorations. Fixed implant-supported prostheses are now preferred for their aesthetic appeal, functional efficiency, and comfort [3]. However, alveolar bone resorption can complicate implant placement in cases of prolonged edentulism. This often necessitates advanced surgical procedures such as grafting, nerve lateralization, or sinus lifting. However, these techniques are associated with risks including infection, graft failure, and nerve injury. Moreover, they may prolong treatment duration, thereby reducing patient comfort. As an alternative, tilted implant placement has achieved success rates comparable to those of conventional axial systems, reducing the need for advanced surgeries [4,5,6,7]. Based on this principle, the All-on-4 concept involves the application of a fixed full-arch prosthesis supported by two straight and two tilted implants in the mandible. The posterior implants are placed at an angle of 30–45° to overcome anatomical limitations, shorten treatment duration, and lower complication risks [8,9,10]. Long-term follow-up studies have reported high implant and prosthesis survival rates using the All-on-4 protocol, with average marginal bone loss of approximately 1.7 mm in 10 years [11].

Angulation of the anterior implants can further enhance prosthesis stability in varying anatomical scenarios and reduce cantilever length. In particular, “V” or “M” configurations applied in patients with atrophic jaws may enhance prosthesis durability and implant stability [12,13].

The variation in mandibular arch form must also be carefully considered in such implant placement approaches. The mandibular arch morphology varies among individuals across different populations and is influenced by factors such as age, sex, and ethnic background. Misch defined three basic arch forms directly influencing implant placement: square, ovoid, and tapered (V-shaped) [14].

In implant biomechanics, not only implant configuration but also surface characteristics are critical. Similar to the evolution of dental restorative materials from polymer-based resins to nano-filled composites, implant surface designs have also undergone significant advancements [15]. Although most contemporary implant designs have micro-roughened surfaces, trabecular implants have three-dimensional porous structures that enable bone to grow both onto and into the implant, promoting a wider distribution of stress and a more balanced load transfer at the bone-implant interface. Compared with conventional micro-roughened implants, trabecular designs aim to reduce stress concentrations and improve long-term stability, which is particularly relevant for managing the high functional loads observed in full-arch fixed prostheses. However, they are associated with challenges such as difficulties in treating peri-implantitis, risk of mechanical fracture in dense bone, and lack of long-term clinical data [16]. Porous tantalum is beneficial in terms of biocompatibility and osseointegration; however, its high density, challenges in processing, and expensive manufacturing limit its application [17]. Given the limitations related to tantalum-based trabecular implants, a titanium design with a selectively porous midsection was developed in this study to preserve biomechanical advantages while addressing known drawbacks.

The primary aim of this study was to design trabecular-surfaced implants and evaluate whether they improve stress distribution compared with standard implants in fixed prosthetic restorations. The secondary aim was to analyze stress distribution patterns under different implant placement configurations and mandibular arch forms. Moreover, the study investigated the advantages and limitations of both implant types and assessed the clinical applicability of the trabecular design across various anatomical conditions.

## 2. Materials and Methods

### 2.1. Ethical Approval

This study was a collaboration between the Faculty of Dentistry at Istanbul University and Ay Tasarım Ltd. Şti. It was supported by the Scientific Research Projects Coordination Unit of Istanbul University (project number: “TDK-2023-40115”). It was approved by the Ethics Committee of the Faculty of Dentistry, Istanbul University (Decision No.: 2023/36).

### 2.2. Mandibular Arch Models and Selection

Digital mandibular models were modified from existing templates using the three-dimensional (3D) design software Blender 4.1 (Blender Foundation, Amsterdam, The Netherlands). The reference models were constructed referring to the study by Bilgin [18], who examined 400 edentulous mandibles from the Turkish population. In that study, the mandibular arch forms were classified as hyperbolic (19.75%), U-shaped (18.75%), and elliptical (1.75%). In this study, the most prevalent hyperbolic and U-shaped forms were selected for modeling. In the study by Bilgin, the length measurements were taken as the distance on the coronal plane passing through two retromolar points in mandibular drawings. Width measurements were defined as the distance between sagittal planes tangent to the vestibular action boundary line. The models selected for the study were as follows:

- Hyperbolic mandibular model: 50 mm anteroposterior length and 75 mm transverse width
- U-shaped mandibular model: 55 mm anteroposterior length and 70 mm transverse width

The elliptical morphology was not included in the model due to its extremely low prevalence. The cortical bone thickness was modeled as 2 mm. The inner part of the cortical bone layer on the outer surface was modeled as cancellous bone.

### 2.3. Implant Configurations

Four different implant configurations were constructed for each mandibular model. All implants were 4.1 mm in diameter and 12 mm in length, maintaining consistent dimensions across all models to ensure geometric and biomechanical uniformity. An angulation of 30° was selected, as it represents one of the most preferred inclinations in clinical All-on-4 applications [4]. Implant placement configurations are summarized in Table 1.

**Figure 1:**
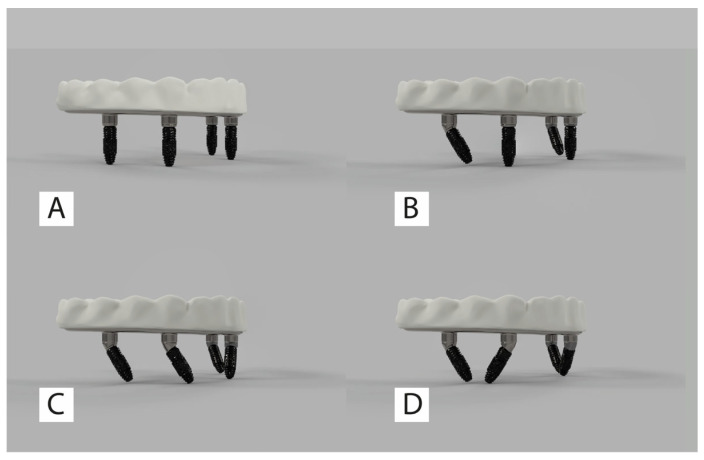
Schematic representation of the four implant placement configurations, with (**A**–**D**) indicating Configurations 1, 2, 3, and 4.

A total of 16 models were created. Figure 1 illustrates the implant placement configurations.

### 2.4. Implant Design

The standard implants used in the study were from the DE2 series (Detech Implant Technology, Ankara, Turkey).

**Figure 2:**
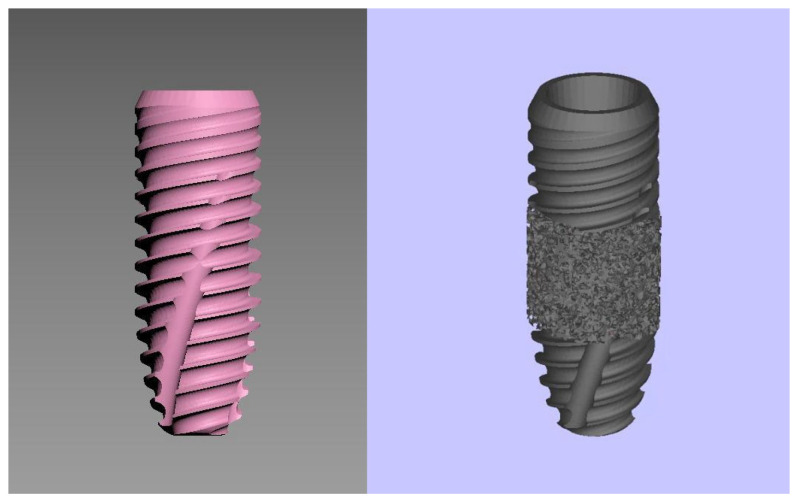
Digital representation of a conventional implant (**left**) and a trabecular implant with porous midsection (**right**).

The trabecular-surfaced implant design was developed in cooperation with the same company. In this design, a trabecular titanium surface structure was applied only to the middle one-third of the implant (average pore size is ~200 μm), whereas the rest retained standard threading. The implants were obtained in stereolithography format from the manufacturer and the prototype production was carried out using Grade 5 titanium (Ti-6Al-4V), modeled in accordance with International Organization for Standardization 5832-3, American Society for Testing and Materials (ASTM) F1472, and ASTM F3302 standards. The differences between trabecular and conventional implants are illustrated in Figure 2.

### 2.5. Prosthesis and Prosthetic Components

The prostheses were digitally designed with a hybrid structure comprising a titanium framework and an acrylic Polymethyl Methacrylate (PMMA) veneering material. The posterior cantilever was designed corresponding to the location of the first molar and cantilever lengths were 23.4 mm in the U-shaped arch and 18.26 mm in the hyperbolic arch (measured from the platform of the distal abutment to the end of the cantilever), the framework had approximate cross-sectional dimensions of 8.5 × 8.5 mm, and a PMMA veneering layer with a uniform thickness of 1.8 mm was applied. Multi-unit abutments and connecting screws were modeled using digital Computer-Aided Design data provided by the manufacturer.

### 2.6. Finite Element Model

All modeling procedures were performed using Blender and Autodesk Fusion 360 (version 2.0.21528). All components were modeled under the assumptions of isotropic, homogeneous, and linearly elastic material properties. Solid mesh types were used to create the finite element meshes. Mesh density was optimized based on solution sensitivity to minimize the dependence of results on mesh size (Table 2). The stress values were determined using nodal probes positioned at consistent landmarks.

Mechanical properties, including elastic modulus and Poisson’s ratio, are detailed in Table 3.

### 2.7. Loading Conditions

A single loading scenario was applied in all models. A 250-N oblique force was directed at a 30° angle to the posterior cantilever region. The aim of this loading condition was to simulate masticatory forces.

### 2.8. Boundary Conditions

**Figure 3:**
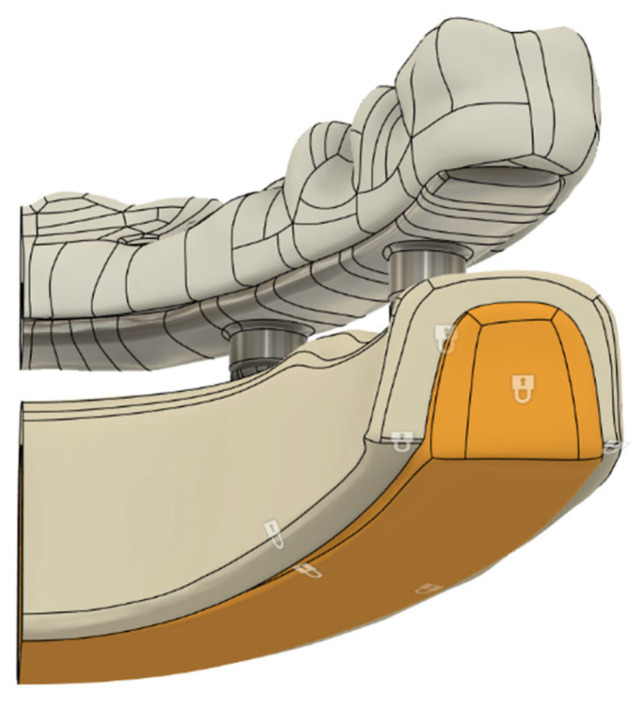
Applied boundary conditions.

Finite element analysis was performed on half-arch mandibular models due to software limitations, particularly to accommodate the high-resolution mesh density required for the trabecular implant design. Simulating the entire mandible with this level of mesh detail would have led to a large number of elements and compromised computational feasibility. The biomechanical continuity of the model was ensured by applying a fixed support condition to the cross-sectional surface generated along the midline plane. Furthermore, the distal ends of the mandibles were fully constrained to stabilize the model during load application. All contact regions between components were defined as bonded, thereby enabling full deformation transfer across implant-bone and prosthetic interfaces (Figure 3).

The closed padlock symbol indicates fully constrained regions (fixed supports).

### 2.9. Group Definitions

All the groups in the study are summarized in Table 4.

## 3. Results

This study evaluated stress distribution under oblique loading in 16 mandibular models created using different arch morphologies (hyperbolic and U-shaped), implant surface types (standard and trabecular), and four different implant configurations.

Table 5 illustrates the maximum and minimum stress magnitudes (in MPa) within cortical and cancellous bone tissues. Among the maximum principal stress (*σ*_1_–tensile) values in cortical bone, U-shaped mandibular models produced lower average stress levels (39.67 MPa) compared with hyperbolic models (45.23 MPa). The maximum principal stress in cortical bone for U-shaped mandibular models was approximately 12.3% lower compared with that for hyperbolic models. Additionally, the lowest maximum principal stress was recorded in trabecular implants (24.75 MPa), whereas the lowest value in the standard implant group was 29.24 MPa.

When comparing implant configurations, Configuration 3 generated the highest tensile stress levels in the cortical bone, with values of 55.832 and 50.227 MPa in the TRH3 and STH3 models, respectively. In contrast, Configurations 2 and 4 generally yielded more moderate stress levels. For instance, the stress values of 43.391 and 42.695 MPa were observed in TRH2 and TRU4, respectively. Trabecular-surfaced implants reduced tensile stress in the cortical bone by approximately 10–30% across configurations. The stress concentration in the cortical bone was mostly seen around implant necks, particularly in Configuration 3 and 4 models, where distally tilted implant placements increased lever arms and led to localized accumulation. These regions were identified by sharp color gradients on tensile and compressive stress maps. On the contrary, Configuration 2 exhibited a more uniform distribution pattern.

Table 6 presents the maximum stress values (in MPa) observed in the implants and prosthetic components. Regarding the von Mises stress at the neck of posterior implants, the highest value was found in the STH1 model (383.382 MPa), whereas the lowest was observed in the TRU4 model (194.959 MPa). The approximately 49.2% difference between these two models highlighted the significant impact of both trabecular surface design and implant configuration on stress reduction in this area. Posterior implant stresses were generally higher in Configurations 1 and 3 and lower in Configurations 2 and 4.

In the neck regions of anterior implants, the highest von Mises stress was recorded in the TRU4 model (239.602 MPa), whereas the lowest was measured in the TRH3 model (92.031 MPa). Configurations 3 and 4 were associated with more focused stress accumulation in the anterior region.

In the prosthesis titanium framework, the highest von Mises stress was observed in the STU1 model with a value of 190.248 MPa, whereas the lowest appeared in the TRH3 model with a value of 127.433 MPa. Models with trabecular-surfaced implants displayed 10–30% lower bar stresses. Hyperbolic models generally produced lower average stress on the bar, although the stress was confined to more limited regions. However, U-shaped models exhibited a more evenly distributed profile.

**Figure 4:**
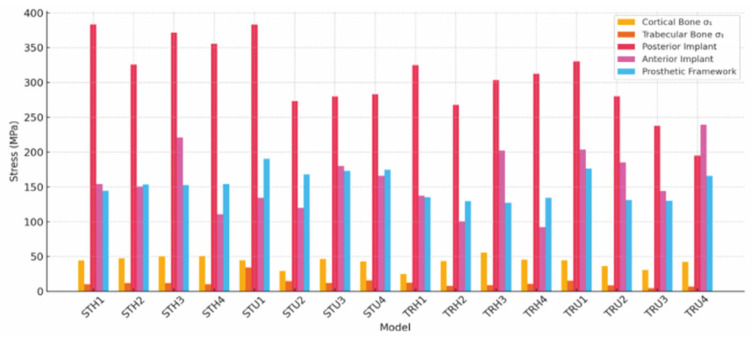
Comparison of stress values among models and components.

Figure 4 provides a comparison of the maximum stress values among all experimental groups. The regional stress analysis indicated that the posterior implant neck regions consistently exhibited the highest concentrations. Stress was more pronounced and localized in models with standard implants, whereas trabecular-surfaced implants facilitated broader and more gradual stress dispersion. In specific cases, such as STU1, the maximum principal stress (*σ*_1_–tensile) in cancellous bone approached or surpassed the critical threshold. This finding was especially evident in configurations combining standard implants with placement pattern 1. Factors such as cantilever effect, limited load sharing, and geometric concentration likely contributed to the observed peaks, potentially increasing the risk of cancellous bone resorption. In contrast, trabecular implants used in combination with U-shaped arches and placement patterns 2–4 exhibited lower compressive stress values and more favorable stress distribution. These configurations helped reduce peak stresses and facilitated more homogeneous load transfer in the trabecular bone.

**Figure 5:**
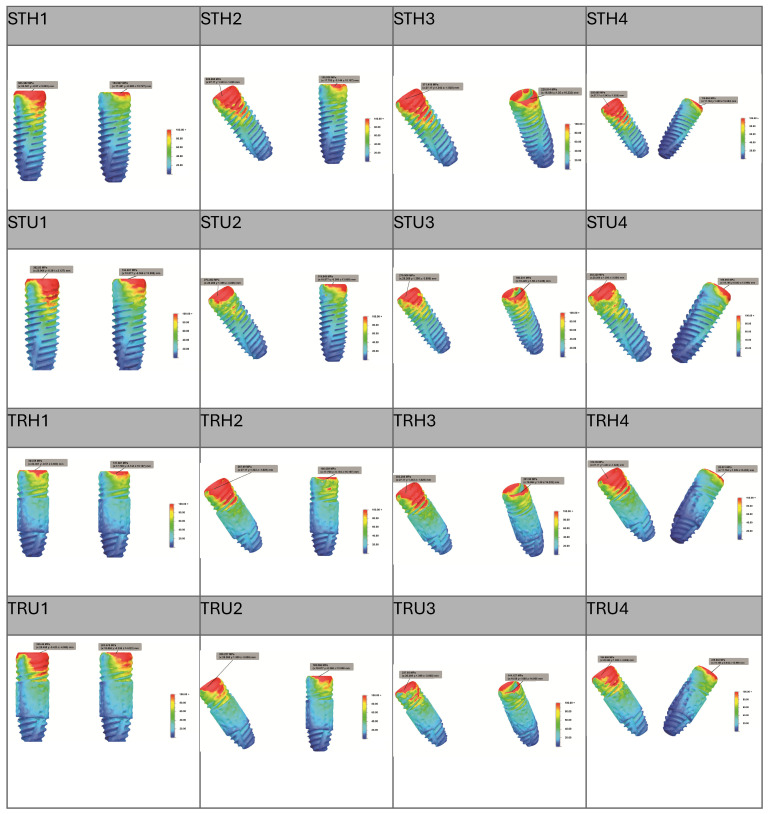
Von Mises stress distribution on implants.

**Figure 6:**
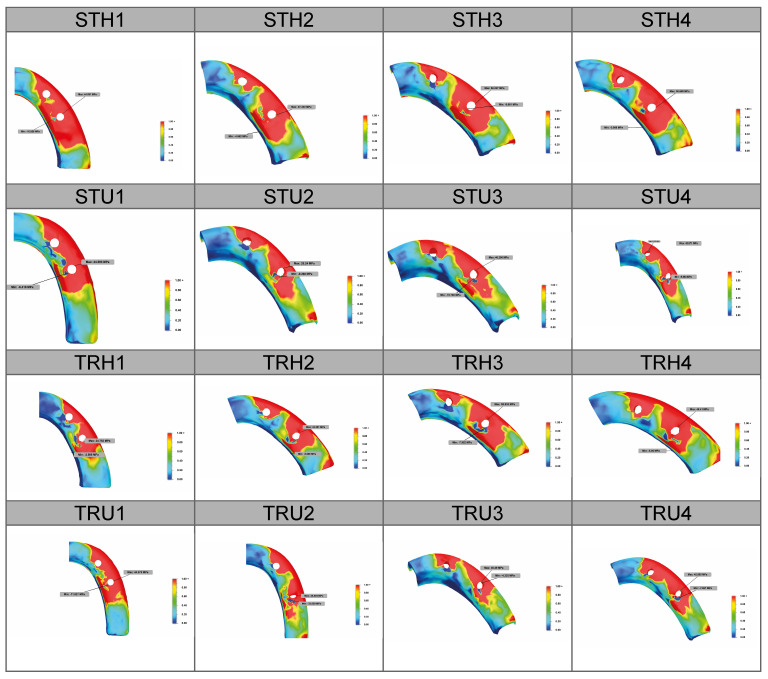
Stress distribution patterns and locations in the cortical bone.

Figure 5 and Figure 6 illustrate the distribution of maximum von Mises stress in the implants and bone across the groups. Overall, the models featuring trabecular-surfaced implants (TRH and TRU) displayed more uniform stress distribution and lower local concentrations. In contrast, standard implant models (STH and STU), especially around posterior implants in cancellous regions, demonstrated intense stress zones near or beyond 10 MPa. This supported the biomechanical relevance of implant surface design in influencing stress patterns.

Stress accumulation was again most prominent around implant necks in the cortical bone. The use of distally inclined implants intensified lever effects, resulting in higher localized stress in these regions, particularly in Configurations 3 and 4. This phenomenon was visible through distinct color shifts in the stress maps. In contrast, Configurations 1 and 2 were associated with a more even distribution. These findings suggest that trabecular-surfaced implants and specific configurations, particularly in U-shaped arches, may offer biomechanical advantages by reducing stress concentrations. These strategies can potentially improve long-term implant stability and minimize bone resorption risks in clinical applications.

## 4. Discussion

This study evaluated combinations of different mandibular arch forms using four implant configurations and two implant surface types in terms of stress distribution under fixed prosthetic structures resembling the All-on-4 concept. According to the finite element analyses, U-shaped arch models generated lower stress values in both cortical and cancellous bone tissues, thus demonstrating biomechanical superiority.

Trabecular-surfaced implants tended to reduce both tensile (1st principal) and compressive (3rd principal) stresses, suggesting that this surface structure enabled more homogeneous load transfer and provided a biomechanically favorable structure. Particularly, Configurations 2 and 4 achieved more balanced load distribution without inducing excessive stress in bone, thus emerging as prominent configurations in this study.

However, the combination of a U-shaped arch, standard implant, and Configuration 1 resulted in the highest tensile stress among all groups and was evaluated as the most biomechanically risky scenario. This outcome might be associated with cantilever effects, limited load transfer, and stress concentration related to arch geometry. Configuration 3 yielded the highest stress values, especially in the cortical bone. Cortical bone stresses above ~50–60 MPa may induce microdamage [21]. In Configuration 3, both TRH3 (55.382 MPa) and STH3 (50.227 MPa) approached this threshold, suggesting a potential biological concern, such as cortical microcracks and bone resorption, which may negatively affect long-term implant stability. This outcome may be attributed to the distal angulation of all implants in Configuration 3, which could have resulted in a more heterogeneous load distribution and stress concentration in the cortical region, compared to the other configurations. Such angulation may also have amplified the cantilever effect, increasing lever moments around the implants and thereby contributing to localized stress accumulation. The geometric form led to stress accumulation in specific regions in hyperbolic arch models, resulting in localized stress peaks.

The highest von Mises stress values under oblique loading were observed in the posterior implants in all groups. This finding highlighted the load-bearing role of posterior implants and their biomechanical criticality. Furthermore, the stress map evaluations revealed that the stress distribution was more localized with sharper transitions in standard implants, whereas it was more widespread and homogeneous in trabecular-surfaced implants. This suggested that the porous structure distributed loads over a broader area, thereby limiting the formation of peak stress zones.

Ayali et al. demonstrated the biomechanical advantages of anteriorly tilted “V” and “M” implant configurations in atrophic maxillae [12]. Similarly, Jensen et al. reported improved prosthetic stability in the mandible using the “V-4” configuration, where anterior implants were tilted distally by approximately 30° [13]. In the present study, Configuration 3 closely mirrored these geometries, incorporating fully distally inclined implants to enhance anteroposterior implant distribution. In our mandibular models, although Configuration 3 did not exceed critical stress thresholds, it generally exhibited higher and more localized stress concentrations compared with other configurations. These results might have stemmed from all implants being tilted in the same direction, causing load concentration in specific areas. Distal angulation can reduce the bone-implant contact surface, leading to an increase in localized stress [22]. The combination of mandibular anatomy and implant angles may lead to uneven stress distribution. Additionally, uniform distal tilting can reduce load sharing among implants and increase lateral forces, resulting in increased stress levels. On the contrary, Salyut et al. [23] also reported no statistically significant difference in marginal bone loss between tilted and axial implants provided that proper surgical planning and appropriate prosthetic restoration were ensured within physiological limits.

The impact of mandibular arch morphology on prosthesis biomechanics has been frequently highlighted in previous studies. Sevinç Gül et al. and Sagat et al. reported that U-shaped arches were advantageous in terms of load distribution, whereas V-shaped arches led to increases in localized stress [24,25]. These findings aligned with the lower stress values observed in U-shaped arches in the present study. The better performance of the U-shaped mandible in this study might be due to several factors. The U-shape allowed for a wider and more balanced load distribution between implants, leading to more homogeneous stress and reduced localized stress concentrations. In contrast, the V-shaped mandible formed sharper angles that caused load concentration in specific areas, increasing stress buildup and cantilever risk [24,25]. Also, Ulu et al. found that increasing the number of implants led to the greatest reduction in tensile stress in the cortical bone for the triangular arch form. The greatest decrease in compressive stress was observed in the square arch under vertical loading, whereas the triangular arch showed the most significant reduction under oblique loading [26].

Numerous studies have demonstrated the biomechanical superiority of trabecular-surfaced implants. Battula et al. reported that the 3D porous structure of these implants promotes a more balanced stress distribution at the bone-implant interface [27]. Bobyn et al. showed that tantalum implants with 75–80% porosity supported bone ingrowth into the implant and accelerated osseointegration [28]. Lee et al. further noted that these structures provided greater secondary stability in individuals with low bone density [29]. However, Bencharit et al. highlighted several drawbacks of these structures, including an increased risk of oral infection, difficulties in cleaning the surface during peri-implantitis treatment, and potential mechanical fragility, despite their strong biological integration [16]. The porous structure of trabecular surfaces promotes bacterial biofilm formation and hinders cleaning, thereby worsening peri-implantitis and complicating treatment. Moreover, the trabecular structure is mainly located in the middle one-third of the implant; therefore, infection may initially progress similarly to that in a normal implant but can advance much more rapidly once it reaches this porous region. The mentioned risk of fracture may exist especially during high-torque placement in dense bone (type 1) and is attributed to the connection structure between Ti and Ta.

The trabecular-surfaced implants used in this study were designed based on previously explored tantalum-based structures but were reengineered using titanium, which is a more biocompatible, mechanically reliable, and easier-to-process material, to eliminate the disadvantages associated with tantalum. This design aimed to preserve the osseointegrative benefits of tantalum implants while addressing production difficulties, cost, and clinical complications. Thus, a more applicable, biomechanically stable, and clinically feasible trabecular surface alternative was developed.

Further, Zhang Ling et al. reported that trabecular implants produced smaller increases in stress compared with titanium implants across three different bone densities [30]. Lingam and Balasubramanian also emphasized the osseointegration potential of porous tantalum, while highlighting its complex and costly production process [17]. In light of these data, the trabecular-surfaced titanium implants used in this study appeared to optimize stress transmission through their high surface area and roughness. The porous architecture of trabecular structures allowed load distribution over a wider area, thus reducing localized stress accumulation. Additionally, bone cells deeply infiltrated the pores, resulting in 3D osseointegration that extended load transfer beyond the surface to the internal structure of the implant [31].

In this study, trabecular-surfaced implants significantly reduced von Mises stress in the posterior regions compared with standard implants. This finding was consistent with the results of Akbarzadeh et al., who reported a 14–32% reduction in stress [32].

Some of the stress values measured in cancellous bone approached the threshold levels reported in previous studies. However, it was presumed that linear elastic models might overestimate stress values in cancellous bone [33,34]. Therefore, such findings should be interpreted cautiously.

The reduction of distal bone support and the more vertical placement of implants led to the transmission of forces over narrower areas in the hyperbolic arch form, resulting in localized stress peaks. In contrast, the broader distribution of implants in the U-shaped arch allowed for more homogeneous load transfer, leading to generally lower stress levels.

The trabecular-surfaced implants were shown to provide more uniform load transfer, limit stress accumulation, and enhance biomechanical balance in all groups, particularly in challenging anatomical scenarios. Additionally, the torque and fatigue tests revealed that these implants possessed high durability and longevity, further supporting their suitability for clinical applications.

However, the present study was limited to finite element analysis. All implant groups were assumed to be fully osseointegrated due to the nature of finite element modeling. The histological comparisons demonstrated that the trabecular-surfaced implants exhibited a greater and more consistent bone-to-implant contact [35]. Therefore, the stress levels may be even lower in the case of trabecular-surfaced implants compared with standard implants in clinical applications. Percentagewise, higher values can be achieved in favor of trabecular-surfaced implants. Additionally, implant length and diameter are clinically important parameters in stress transfer. Longer implants can increase the contact area with cortical bone and contribute to a wider distribution of loads, while wider implants can improve load transfer and reduce local stress peaks. In contrast, shorter or narrower implants may increase stress concentrations, particularly in cases with limited bone support [36]. Further advanced modeling and in vivo studies are required to validate the findings of this study and compare the proposed design with similar implant systems.

This study offers valuable biomechanical insights into All-on-4 full-arch implant-supported prostheses, despite assumptions such as isotropic, homogeneous, and linearly elastic material properties; evaluation under a single oblique loading scenario; the assumption of full osseointegration; and other simplifications inherent to finite element analysis.

## 5. Conclusions

Based on the findings obtained under oblique loading conditions, this study demonstrated that the combination of mandibular arch shape, implant surface characteristics, and implant configuration significantly influenced stress distribution on both bone and implant structures. The U-shaped mandibular model was found to be biomechanically more advantageous, producing lower stress levels in both cortical and cancellous bones. Trabecular-surfaced implants facilitated a more balanced load transfer by distributing stress over a broader area.

Clinical recommendations are as follows:

- Trabecular-surfaced implants may be preferred in cases with a hyperbolic arch form due to their superior ability to reduce localized stress concentrations, promote more favorable load transfer, and potentially minimize biological complications such as cortical microdamage and bone resorption; if possible, Configuration 2 or 4 should be selected, while Configuration 3 approached critical stress thresholds and may involve higher biological risks.
- The high stress levels observed in posterior implants indicate that this region should be prioritized during clinical planning. The primary reason for the higher von Mises stresses observed in posterior implants is that these regions are subjected to greater masticatory forces, with occlusal loads typically reaching their maximum in the posterior areas. Since such stresses can be reduced by distributing load transfer over a larger surface area, it is recommended in clinical practice to prefer the longest and widest implants possible within clinical limits. In patients with higher masticatory forces, the stress-reducing and load-distributing effects of trabecular-surfaced implants may become more clinically relevant. Additionally, in such patients, supplementary strategies such as increasing the number of implants or selecting alternative prosthetic materials may also be considered.
- Configuration 2 emerged as the most favorable configuration in terms of balanced load transfer and low stress levels; however, Configurations 3 and 4 may serve as effective alternatives in anatomically constrained cases.

These results highlight the importance of individualizing implant positioning and surface selection based on mandibular morphology to ensure clinical success. To further validate the clinical applicability of these findings, long-term prospective clinical studies and in vivo biomechanical assessments, such as radiographic evaluations of marginal bone loss, are required.

## Figures and Tables

**Table 1:** Implant placement configurations.

Configuration	Implant 1	Implant 2	Implant 3	Implant 4
Configuration 1	0°	0°	0°	0°
Configuration 2	30° distal	0°	0°	30° distal
Configuration 3	30° distal	30° distal	30° distal	30° distal
Configuration 4	30° distal	30° mesial	30° mesial	30° distal

**Table 2:** Mesh characteristics of four representative models selected based on arch type, implant configuration, and implant surface.

Group Code	Implant Type	Arch Type	Configuration	Node Count	Element Count
STH4	Standard	Hyperbolic	Configuration 4	263,606	1,368,172
TRH2	Tr. surfaced	Hyperbolic	Configuration 2	329,923	1,746,265
STU1	Standard	U-shaped	Configuration 1	222,738	1,134,275
TRU3	Tr. surfaced	U-shaped	Configuration 3	343,629	1,832,555

**Table 3:** Poisson’s ratio and Young’s modulus values based on the Finite Element Method.

Material	Young’s Modulus (MPa)	Poisson’s Ratio
Ti-6Al-4V	113,763	0.35
PMMA	2740	0.35
Titanium	102,800	0.36
Cancellous bone	1370	0.30
Cortical bone	13,000	0.30

Based on data from Refs. [19,20].

**Table 4:** Detailed description of the study groups.

Group Name	Arch Type	Configuration	Implant Type
STH1	Hyperbolic	Configuration 1	Standard
STH2	Hyperbolic	Configuration 2	Standard
STH3	Hyperbolic	Configuration 3	Standard
STH4	Hyperbolic	Configuration 4	Standard
STU1	U-shaped	Configuration 1	Standard
STU2	U-shaped	Configuration 2	Standard
STU3	U-shaped	Configuration 3	Standard
STU4	U-shaped	Configuration 4	Standard
TRH1	Hyperbolic	Configuration 1	Trabecular-surfaced
TRH2	Hyperbolic	Configuration 2	Trabecular-surfaced
TRH3	Hyperbolic	Configuration 3	Trabecular-surfaced
TRH4	Hyperbolic	Configuration 4	Trabecular-surfaced
TRU1	U-shaped	Configuration 1	Trabecular-surfaced
TRU2	U-shaped	Configuration 2	Trabecular-surfaced
TRU3	U-shaped	Configuration 3	Trabecular-surfaced
TRU4	U-shaped	Configuration 4	Trabecular-surfaced

**Table 5:** Cancellous and cortical bone stress distributions (MPa).

Group	Cortical Max σ1 (MPa)	Cortical Min σ3 (MPa)	Cancellous Max σ1 (MPa)	Cancellous Min σ3 (MPa)
STH1	44.097	−70.26	10.201	−5.772
STH2	47.433	−88.425	11.73	−9.399
STH3	50.227	−70.642	11.709	−9.659
STH4	50.658	−76.729	10.278	−9.553
STU1	44.593	−70.761	34.594	−9.704
STU2	29.24	−71.386	14.533	−5.365
STU3	46.296	−82.069	11.824	−6.501
STU4	42.871	−75.639	15.78	−6.678
TRH1	24.752	−55.689	12.364	−7.023
TRH2	43.391	−57.731	8.271	−7.625
TRH3	55.382	−73.635	0.877	−9.829
TRH4	45.41	−57.747	10.556	−8.238
TRU1	44.575	−61.401	15.453	−10.205
TRU2	36.604	−73.719	8.568	−7.322
TRU3	30.49	−60.419	4.768	−4.371
TRU4	42.695	−62.039	6.922	−5.044

**Table 6:** Maximum von Mises stress distributions for components.

Group	Implant Ant. (MPa)	Implant Post. (MPa)	Framework VM (MPa)
STH1	153.927	383.382	144.315
STH2	150.936	325.555	153.419
STH3	220.914	371.418	152.383
STH4	110.852	355.695	153.961
STU1	134.401	382.83	190.248
STU2	119.949	273.372	167.762
STU3	180.231	279.906	172.729
STU4	166.093	283.229	174.661
TRH1	137.687	324.55	135.095
TRH2	100.559	267.999	129.467
TRH3	201.9	303.208	127.433
TRH4	92.031	312.39	134.159
TRU1	203.678	330.45	176.16
TRU2	185.666	280.227	130.778
TRU3	144.127	237.93	130.022
TRU4	239.602	194.959	165.714

## Data Availability

Raw data supporting the results of this study are available from the authors upon request.
